# Evidence for Non-Essential Salt Bridges in the M-Gates of Mitochondrial Carrier Proteins

**DOI:** 10.3390/ijms23095060

**Published:** 2022-05-02

**Authors:** Daniela Valeria Miniero, Magnus Monné, Maria Antonietta Di Noia, Luigi Palmieri, Ferdinando Palmieri

**Affiliations:** 1 Department of Biosciences, Biotechnologies and Biopharmaceutics, University of Bari, Via E. Orabona 4, 70125 Bari, Italy; danielavaleria.miniero@uniba.it (D.V.M.); magnus.monne@unibas.it (M.M.); maria.dinoia@uniba.it (M.A.D.N.); 2 Department of Sciences, University of Basilicata, Via Ateneo Lucano 10, 85100 Potenza, Italy; 3 CNR Institute of Biomembranes, Bioenergetics and Molecular Biotechnologies (IBIOM), 70126 Bari, Italy

**Keywords:** GDP/GTP carrier, membrane protein, mitochondrial carrier, NAD^+^ carrier, oxoglutarate carrier, transport mechanism, site-directed mutagenesis, solute carrier family 25 SLC25, substrate-transporter interactions

## Abstract

Mitochondrial carriers, which transport metabolites, nucleotides, and cofactors across the mitochondrial inner membrane, have six transmembrane α-helices enclosing a translocation pore with a central substrate binding site whose access is controlled by a cytoplasmic and a matrix gate (M-gate). The salt bridges formed by the three PX[DE]XX[RK] motifs located on the odd-numbered transmembrane α-helices greatly contribute to closing the M-gate. We have measured the transport rates of cysteine mutants of the charged residue positions in the PX[DE]XX[RK] motifs of the bovine oxoglutarate carrier, the yeast GTP/GDP carrier, and the yeast NAD^+^ transporter, which all lack one of these charged residues. Most single substitutions, including those of the non-charged and unpaired charged residues, completely inactivated transport. Double mutations of charged pairs showed that all three carriers contain salt bridges non-essential for activity. Two double substitutions of these non-essential charge pairs exhibited higher transport rates than their corresponding single mutants, whereas swapping the charged residues in these positions did not increase activity. The results demonstrate that some of the residues in the charged residue positions of the PX[DE]XX[KR] motifs are important for reasons other than forming salt bridges, probably for playing specific roles related to the substrate interaction-mediated conformational changes leading to the M-gate opening/closing.

## 1. Introduction

Mitochondrial carriers (MCs) are a superfamily of transporters that in higher animals is called the solute carrier family 25 (SLC25) [1,2,3,4,5]. With a few exceptions, the MCs are localized in the inner mitochondrial membrane where they transport various substrates, such as nucleotides, amino acids, metabolites, and cofactors, across the membrane [6,7,8,9]. The substrates of the majority of the 53 MC family members in *Homo sapiens*, 58 in *Arabidopsis thaliana,* and 35 in *Saccharomyces cerevisiae* have been identified by the EPRA method, in which MCs are expressed recombinantly, purified, and reconstituted into liposomes for transport assays [6,10,11]. Examples of MCs are the ADP/ATP carrier (AAC), the oxoglutarate carrier (OGC), the GTP/GDP carrier (Ggc1p), and the NAD^+^ transporter (Ndt1p) [10,12,13,14,15]. The EPRA approach has also been applied to investigate other transport properties, such as kinetic parameters, uniport, antiport, or symport modes of transport as well as to characterize variants of MCs, among which many are associated with several human genetic diseases [3,16,17,18,19].

Characteristic sequence motifs are hallmarks of MCs, which all consist of triple 100-residue sequence repeats, each containing two transmembrane segments connected by a signature motif sequence PX[DE]XX[KR]X[KR]X_20-30_[DE]GXXXX[WYF][KR]G (PROSITE PS50920, PFAM PF00153, and IPR00193) [20,21]. In the mitochondrial inner membrane, the six transmembrane segments of MCs assume a topology with the N- and C-termini in the intermembrane space [22,23,24,25]. In the 3D structures of AAC, the hydrophobic segments form a bundle of six transmembrane α-helices (H1–H6), which surround a central substrate translocation pore with two gates: one towards the mitochondrial matrix (M-gate) and one towards the intermembrane space (or cytoplasm, C-gate) [26,27,28]. In the structures of the carboxyatractyloside-inhibited bovine AAC1 (bAAC1) and yeast AACs, the inhibitor is bound in the central translocation pore with the C-gate open and the M-gate closed in a conformation that is thought to correspond approximately to the C-state of the carrier, ready to receive a substrate from the intermembrane space (cytoplasm) side of the membrane [26,27]. In these structures, the prolines of the three signature motifs make a kink in the odd-numbered α-helices, and the following two charged residues form a salt bridge network interconnecting H1, H3, and H5 as part of the closed M-gate [26,27,29,30]. In contrast, the structure of the bongkrekic acid-inhibited *Thermothelomyces thermophila* AAC (TtAAC) resembles the M-state, in which the M-gate is open with its salt bridge network broken and the C-gate is closed, essentially by another salt bridge network formed by the less conserved [FY][DE]XX[KR] motifs of the even-numbered α-helices [28,31]. The gates are alternating between being open and closed, and located between them in the central translocation pore, approximately at the level where the inhibitors bind, is the substrate binding site, which is formed mainly by the residues of the so-called contact points (I, II, and III) in H2, H4, and H6 [32]. Based on the characterized transport properties, sequence, and structural features, a common transport mechanism for MCs has been suggested [33,34,35,36,37]: the substrate enters through the open gate and binds to the centrally-located substrate binding site, the binding energy triggers conformational changes leading to the closing of the open gate and opening of the closed gate. Subsequently, the substrate exits on the opposite side of the membrane with respect to where it entered, and the carrier is ready to transport another substrate in the opposite direction. Interactions of the substrate with the contact point residues on H2, H4, and H6, and other residues exposed towards the substrate binding site, may all contribute to the binding energy driving the carrier conformational changes [4,15,32,35,38,39].

Until now, it has been generally thought that the charged residues of the PX[DE]XX[KR] motifs located on the odd-numbered transmembrane α-helices (at the beginning of the signature motif sequences as defined above, or being the MC signature motif sequence themselves as indicated in some publications) are important for their ability to form salt bridges between the helices H1, H3, and H5 and, thereby, for closing and opening the M-gates of the MCs. However, out of the 53 human MCs, 12 miss one charged residue and 10 miss even more charged residues (between two and five). This rather underestimated fact shows that the complete salt bridge network of the M-gate is not a feature present in all members of the MC family. Furthermore, the importance of the substitutions of the charged residues as well as the relevance of each charged residue in the PX[DE]XX[KR] motifs of the MCs have been scarcely studied and are still poorly understood. 

In this work, we have investigated the functional role(s) of the charged residue positions in the PX[DE]XX[KR] motifs of MCs that have incomplete salt bridge networks by lacking one of their three salt bridges: OGC (not having the H1–H3 salt bridge), Ggc1p (lacking the H1–H5 salt bridge), and Ndt1p (missing the H3–H5 salt bridge). Both single and double cysteine mutations of the PX[DE]XX[KR] motif charged residues in Ggc1p and Ndt1p (which have not been investigated previously in this respect) and double cysteine substitutions in OGC (in which single mutations of the above residues have been studied before) were introduced by site-directed mutagenesis. Their transport activities measured by the EPRA method show, for the first time, that (i) not all salt bridges of the M-gate are essential for activity and (ii) some of the residues in the charged residue positions of the PX[DE]XX[KR] motifs are important for reasons other than forming salt bridges, probably for interactions occurring in the substrate-induced transition of the M-gate from the closed to the open state, or vice versa.

## 2. Results

### 2.1. Structure and Sequence Analysis of the Matrix Salt Bridge Network of MCs

**Figure 1:**
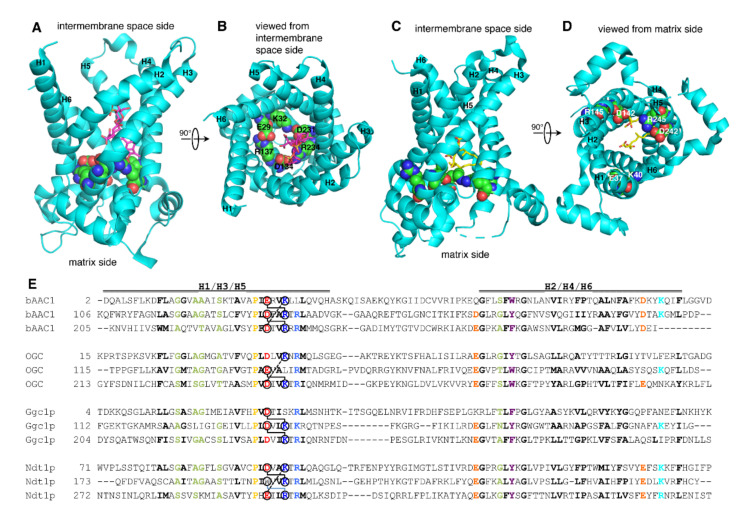
Structure and sequence analysis of the matrix charged network of MCs. Bovine AAC1 structure (cyan) with carboxyatractyloside (sticks with carbons in magenta) and the charged residues of the PX[DE]XX[KR] motifs (in spheres) viewed from the membrane plane (**A**) and the intermembrane space side (**B**). TtAAC structure (cyan) with bongkrekic acid (sticks with carbons in yellow) and the charged residues of the PX[DE]XX[KR] motifs (in spheres) viewed from the membrane plane (**C**) and the matrix side (**D**). Sequence comparison of the three mitochondrial carrier domain repeats of bAAC1, OGC, Ggc1p, and Ndt1p (**E**). The following sequence features are indicated: the position of the transmembrane helices H1–H6 (a line above the sequences), and the residues that are conserved (bold); hydrophobic (black); small (green); proline (yellow); negatively charged (red) and positively charged (blue) of the PX[DE]XX[KR] motifs; aromatic (purple); negatively charged (orange) and positively charged (cyan) residues of the even-numbered α-helices. The salt bridge charged pairs of the PX[DE]XX[KR] motifs are indicated by lines.

The interactions of the charged residues of the PX[DE]XX[KR] motifs were analyzed in the X-ray crystal structures of AAC in the C-state (bAAC1) and the M-state (TtAAC) [26,28]. In the bAAC1 structure, carboxyatractyloside is bound approximately at the level of the substrate binding site in the substrate translocation pore, and the interactions of the PX[DE]XX[KR] motifs take part in closing the M-gate, whereas the C-gate is open (Figure 1A) [26]. The network formed by the three PX[DE]XX[KR] motifs consists of E29 of H1 (H1:E29) binding H3:R137, H3:D134 binding H5:R234, and H5:D231 binding H1:K32 (Figure 1B) [26]. It is worth noting that the side chains of the charged residues of the PX[DE]XX[KR] motif make additional H-bonds: H1:E29 with N76 of H2; H3:R137 with backbone R71 of H2; H3:D134 (salt bridge) with R79 of H2; H5:R234 with Y131 of H3; and H5:D231 (salt bridge) with R235 of H5. These residues are conserved in the AAC subfamily and many of their interactions are found in the yeast carboxyatractyloside-inhibited AAC structures as well [27]. In addition, in the yeast AAC structures, a less conserved glutamine, in the fourth position after the first PX[DE]XX[KR] motif, interacts with both the positively and negatively charged residues of the H5–H1 salt bridge in the so-called glutamine brace [27]. Furthermore, H5:D231 and H5:R234 make H-bonds with carboxyatractyloside. Thereby, the PX[DE]XX[KR] salt bridge network interconnects the odd-numbered transmembrane α-helices, and additional specific interactions also connect them to at least H2. In the TtAAC structure, bongkrekic acid is bound approximately at the level of the substrate binding site, the M-gate is partially open, and the C-gate is closed (Figure 1C). In this conformation, the salt bridge network of the PX[DE]XX[KR] motifs, i.e., the M-gate, is dissolved because the odd-numbered α-helices have moved further apart (Figure 1D). Only the intrahelical interactions H1:K40 with Q44 of H1, and H5:D242 (salt bridge) with H5:R245 are present, whereas no interhelical interactions are detected. In conclusion, the charged residues of the PX[DE]XX[KR] motifs in the ADP/ATP carrier, and presumably in all other members of the MC family, function mainly in forming the salt bridge network in the C-state and do not have any clear role in the M-state [26,28].

We have chosen to investigate the functional relevance of the charged residues of the PX[DE]XX[KR] motif in MCs (OGC, Ggc1p, and Ndt1p) that do not have a complete salt bridge network due to the lack of one the charged residues: OGC lacks the salt bridge between H1 and H3, Ggc1p lacks the one between H1 and H5, and Ndt1p misses the one between H3 and H5 (Figure 1E). Structural homology models of OGC, Ggc1p, and Ndt1p in the C- and M-states were generated based on the above-mentioned AAC structures [26,27,28]. In the C-state models of OGC, Ggc1p, and Ndt1p, the charged residues of the PX[DE]XX[KR] motifs form two salt bridges instead of three, as in the AAC structures. Furthermore, in the OGC, Ggc1p, and Ndt1p homology models, none of the additional interactions of the PX[DE]XX[KR] charged residues found in the bAAC and yeast AAC structures were observed because none of the above-mentioned interacting residues are conserved in OGC, Ggc1p, or Ndt1p, with the exception of OGC:R90 (corresponding to bAAC:R79). Furthermore, although all three of these carriers have many of the glutamines in the sequence positions of the glutamine brace (Figure 1E), none of them formed interactions with the proximal salt bridge residues in the three homology models. However, it cannot be excluded that these glutamines make interhelical interactions between the odd-numbered α-helices with the negatively charged residues of the PX[DE]XX[KR] motifs of these MCs in vivo. In addition, in the M-state models of OGC, Ggc1p, and Ndt1p, the side chains of the charged residues of the PX[DE]XX[KR] motifs did not form any obvious interactions.

### 2.2. Effects on Activity by Mutating the Charged Residue Positions in the PX[DE]XX[KR] Motifs of OGC

**Figure 2:**
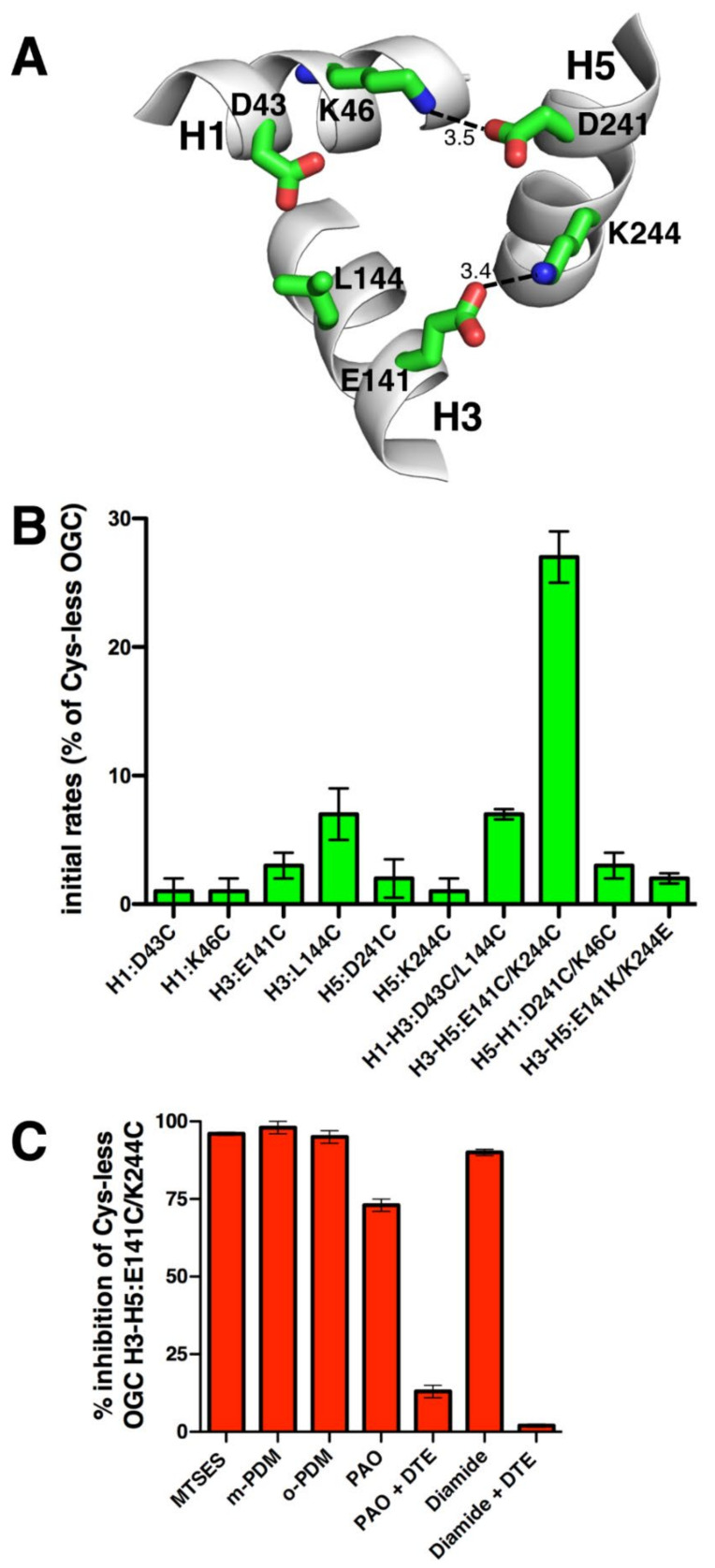
Initial transport rates of the OGC cysteine mutants. (**A**) The PX[DE]XX[KR] motifs in the C-state OGC homology model with the salt bridges indicated by lines and distances in Å. (**B**) Initial transport rates of Cys-less OGC cysteine mutant proteins reconstituted in liposomes. Transport was started by adding 3 mM ^14^C-labelled 2-oxoglutarate to proteoliposomes containing 20 mM 2-oxoglutarate and stopped after 30 sec by the stop inhibitor method. The activity is expressed as the percentage initial rate of Cys-less OGC, which was 2135(±248) μmol/min/g protein. The means ± S.D. of at least three independent experiments carried out in duplicate are shown. The results for the single OGC cysteine mutants have been published previously [30]. (**C**) Liposomes reconstituted with OGC H3-H5:E141C/K244C were treated with the indicated sulfhydryl reagents for 10 min at 25 °C before the transport activity was measured. The inhibition percentages of the initial rate of Cys-less OGC H3-H5:E141C/K244C, which was 568(±127) μmol/min/g protein, are given.

In previous studies, all the residues of the Cys-less OGC have been replaced by cysteines one by one, expressed in *Escherichia coli*, purified from inclusion bodies, and reconstituted into liposomes, which have been used in transport activity assays through the EPRA method [30,40,41,42]. The results showed that substitutions of the charged PX[DE]XX[KR] residues with cysteine H1:D43C, H1:K46C, H3:E141C, H5:D241C, and H5:K244C diminish the initial rate of [^14^C]oxoglutarate/oxoglutarate exchange to less than 4% of the Cys-less OGC activity. However, H3:L144C, the leucine of which is in the position normally occupied by a positively charged residue, decreases the carrier activity to about 7% of the Cys-less OGC (Figure 2A,B) [30]. In the present study, double cysteine-mutants of the charge-pair positions of the PX[DE]XX[KR] motifs in Cys-less OGC were generated and their transport activities were assessed (Figure 2A,B). The H1-H3:D43C/L144C mutant displayed an activity level (7%) comparable to that of the single mutant H3:L144C and slightly higher than that of H3:D43C, which was 1% of the Cys-less OGC activity. Likewise, the transport rate of the H5-H1:D241C/K46C mutant was similar to the very low activities of the corresponding single mutants, showing that this salt bridge is essential for activity. In sharp contrast, H3–H5:E141C/K244C displayed much higher activity (25%) than its single mutants (having 1–3% of the Cys-less OGC activity), strongly suggesting that this salt bridge is not essential for transport, whereas leaving an unpaired charged residue is very detrimental for transport. Furthermore, the mutant with the charged residues swapped between the helices, H3-H5:E141K/K244E, displayed very low activity (2%), indicating that the localization of the paired charged residues within the protein is also important.

The transport activity of the OGC H3-H5:E141C/K244C mutant was further characterized by using sulfhydryl reactive reagents: the membrane-impermeant probe (2-sulfonatoethyl)-methanethiosulfonate (MTSES); the two rigid bi-functional bis-maleimide reagent cross-linkers of cysteines N,N′1,3-phenylenedimaleimide (m-PDM) and N,N′-1,2-phenylenedimaleimide (o-PDM); the close cysteines stable complex-forming phenylarsine oxide (PAO); and the SH-oxidizing agent stimulating disulfide formation diamide (Figure 2C). MTSES, m-PDM, and o-PDM almost completely inhibited the activity of the mutant (90–98% inhibition). Due to the inactivity of the corresponding single mutants of the double H3-H5:E141C/K244C mutant, we were unable to establish if these reagents react with one or both cysteines, or if m-PDM and o-PDM cross-link them. PAO inhibited H3-H5:E141C/K244C to 73%, and the reducing agent dithioerythritol (DTE) reversed its inhibition (Figure 2C). PAO does not react with monothiols but cross-links cysteines that are relatively close together [43]. Similarly, the oxidation agent diamide, which is thought to induce the disulfide bond formation of proximal cysteines [44], inhibited the double cysteine mutant by 90% and its reaction was counteracted by DTE addition. It is noteworthy that the latter inhibitory effects are caused by the reactivity of the reagents and not merely by their presence interfering with substrate binding, as demonstrated by the rescued activity upon the addition of a reducing agent. These results demonstrate that the introduced cysteines in the H3-H5:E141C/K244C mutant are accessible to the above-mentioned sulfhydryl reagents, which, upon reaction with them, block transport. Interestingly, the inhibition by MTSES, which is impermeable to the membrane [45], shows that the introduced cysteines in the OGC H3-H5:E141C/K244C mutant are exposed to the pore cavity of the carrier (accessible to water) and therefore towards the substrate which binds approximately in the middle of the cavity.

### 2.3. Effects on Activity by Mutating the Charged Residue Positions in the PX[DE]XX[KR] Motifs of Ggc1p

**Figure 3:**
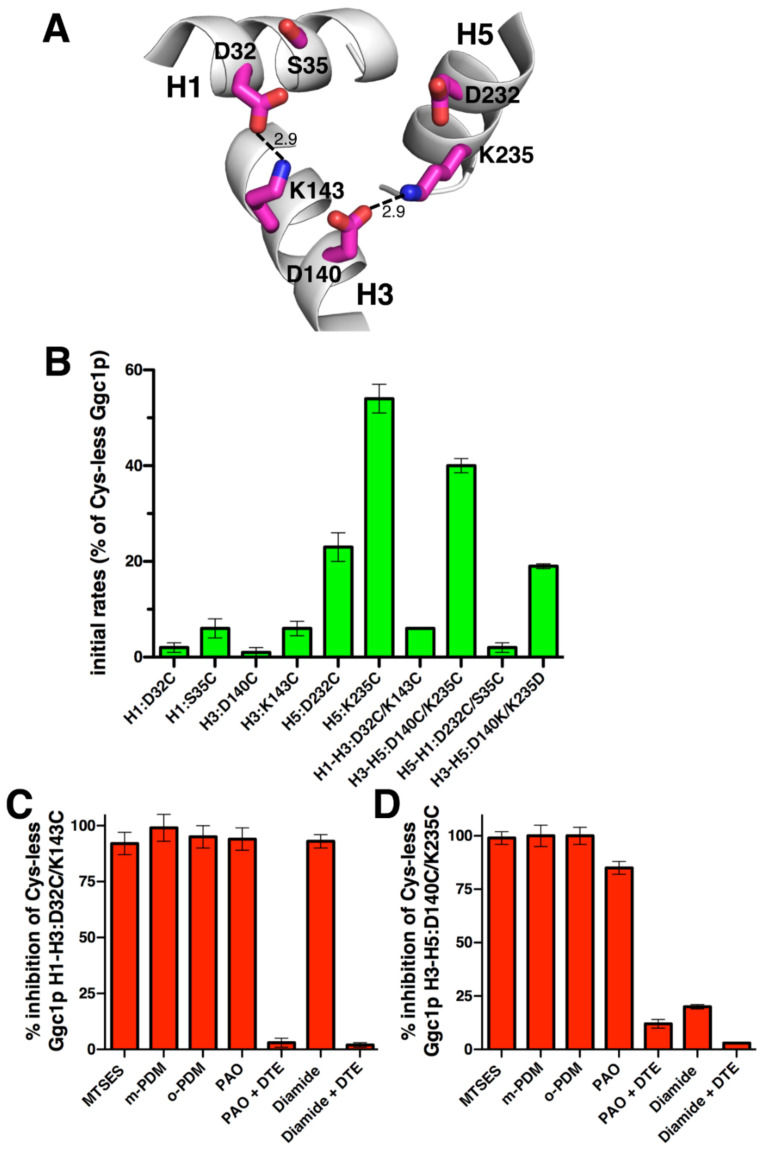
Initial transport rates of the Ggc1p cysteine mutants. (**A**) The PX[DE]XX[KR] motifs in the C-state Ggc1p homology model with the salt bridges indicated by lines and distances in Å. (**B**) Initial transport rates of Cys-less Ggc1p cysteine mutant proteins reconstituted in liposomes. Transport was started by adding 100 μM of ^3^H-GTP to proteoliposomes containing 5 mM GTP and stopped after 30 sec by the stop inhibitor method. The activity is expressed as the percentage initial rate of Cys-less Ggc1p, which was 35678 (±672) μmol/min/g protein. The means ± S.D. of at least three independent experiments carried out in duplicate are shown. Liposomes reconstituted with Cys-less Ggc1p H1-H3:D32C/K143C (**C**) and H3-H5:D140C/K235C (**D**) were treated with the indicated sulfhydryl reagents for 10 min at 25 °C before the transport activity was measured. The inhibition percentages of the initial rate of Cys-less Ggc1p H1-H3:D32C/K143C and H3-H5:D140C/K235C, which were 3367(±126) μmol/min/g protein and 9641(±365) μmol/min/g protein, respectively, are given.

Ggc1p, which was identified and characterized by the EPRA method [13], lacks the salt bridge H1–H5 (Figure 3A). In the present study, we have generated a Cys-less variant of Ggc1p displaying 100% [^3^H]GTP/GTP homoexchange transport activity of the wild-type protein. The charged residue positions in the PX[DE]XX[KR] motifs of Ggc1p were then mutated into cysteines, and the transport rates of the mutant proteins were measured (Figure 3B). H1:D32C, H3:D140C, and H3:K143C as well as H1:S35C exhibited less than 7% activity of that of the Cys-less Ggc1p, whereas H5:D232C and H5:K235C were about 23% and 54% active compared to Cys-less Ggc1p, respectively. The double charge-pair mutant H1-H3:D32C/K143C displayed an activity level almost negligible and equal to that of the single mutant H3:K143C and slightly higher than that of H1:D32C, indicating that this salt bridge is essential for transport. H3-H5:D140C/K235C showed 40% activity, i.e., in between H3:D140C and H5:K235C, suggesting that this salt bridge is not essential for transport. Furthermore, H5-H1:D232C/S35C was as inactive as H1:S35C. H1:S35, in a position that is usually occupied by a positively charged residue, is apparently crucial for activity. When its unpaired charged residue H5:D232 was mutated into cysteine, 23% of activity remained (as compared to Cys-less Ggc1p), indicating that the negatively charged residue in this position is of functional importance but is not strictly necessary. The fact that the H3-H5:D140C/K235C and H5:K235C mutants work quite well, but not H3:D140C, suggests that leaving K235 unpaired is detrimental to transport activity. In addition, exchanging the position of the charged residues of this pair as in the H3-H5:D140K/K235D mutant led to 19% activity compared to Cys-less Ggc1p, showing (again) that the localization of the paired charged residues within the protein is also important.

The effects of the sulfhydryl reactive reagents were tested on the transport activity of the two double cysteine mutants with sufficient activity H1-H3:D32C/K143C and H3-H5:D140C/K235C (Figure 3C,D). Both double mutants were almost completely inhibited by MTSES, m-PDM, and o-PDM (92–100% inhibition), suggesting that these reagents react with at least one of the cysteines, but which one could not be distinguished because one of their corresponding single mutants is totally inactive. PAO and diamide inhibited H1–H3:D32C/K143C to 94% and 93%, respectively, and H3-H5:D140C/K235C to 85% and 20%, respectively. Moreover, PAO and diamide inhibition was reversed by DTE. Taken together, these results indicate that (i) the above-mentioned cysteines of Ggc1p are accessible to several sulfhydryl reagents that inhibit transport activity, (ii) PAO reacts reversibly with both cysteines in H1–H3:D32C/K143C and H3–H5:D140C/K235C mutants, and (iii) diamide reacts with both cysteines only of the former mutant.

### 2.4. Effects on Activity by Mutating the Charged Residue Positions in the PX[DE]XX[KR] Motifs of Ndt1p

**Figure 4:**
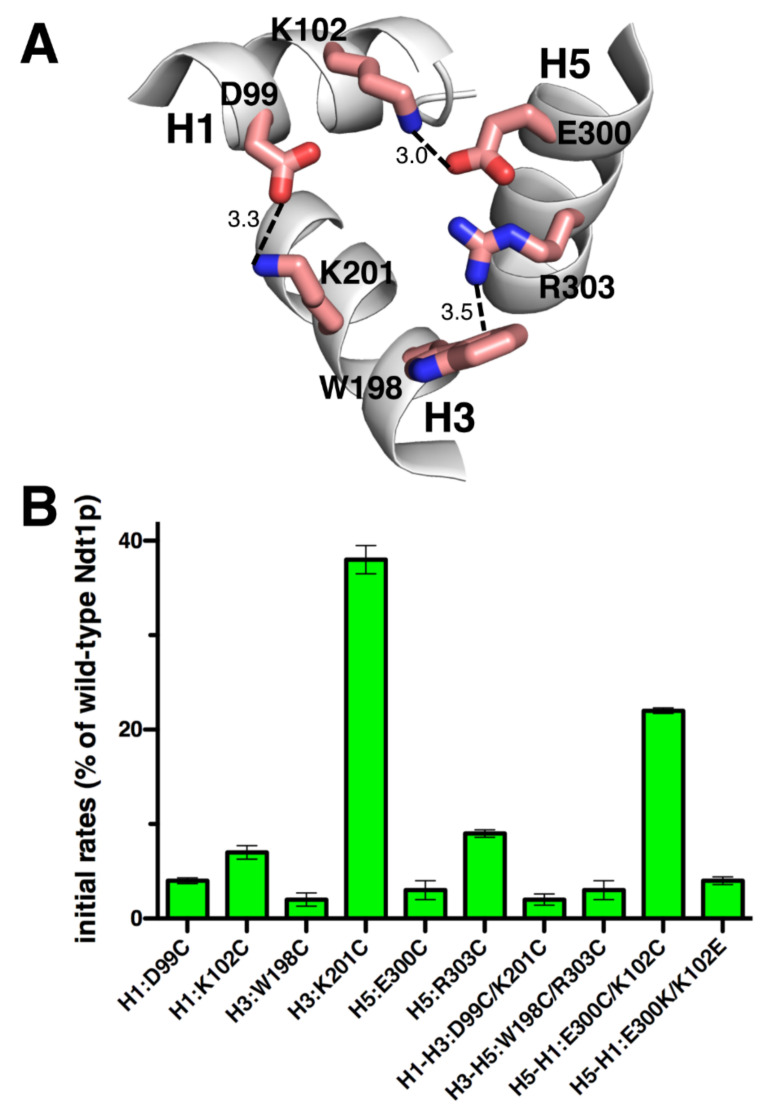
Initial transport rates of the Ndt1p cysteine mutants. (**A**) The PX[DE]XX[KR] motifs in the C-state Ndt1p homology model with the salt bridges and the hypothetical cation-π interaction indicated by lines and distances in Å. (**B**) Initial transport rates of Ndt1p cysteine mutant proteins reconstituted in liposomes. Transport was started by adding 3 mM of ^14^C-NAD^+^ to proteoliposomes containing 20 mM NAD^+^ and stopped after 30 sec by the stop inhibitor method. The activity is expressed as the percentage initial rate of wild-type Ndt1p, which was 1067(±123) μmol/min/g protein. The means ± S.D. of at least three independent experiments carried out in duplicate are shown.

Ndt1p, which is one of the two mitochondrial NAD^+^ transporters of *S. cerevisiae* characterized with the EPRA method [14], lacks the H3 PX[DE]XX[KR] motif negatively charged residue, being replaced by a tryptophan (Figure 4A). In the case of Ndt1p, the cysteine mutants of the charged residues of the matrix gate were generated in the wild-type protein given that upon substitution of all its five cysteines, the carrier transport activity was almost abolished (data not shown). Subsequently, the initial [^14^C]NAD^+^/NAD^+^ homoexchange transport rates of the cysteine mutants made in wild-type Ndt1p were measured (Figure 4B). H1:D99C, H1:K102C, H3:W198C, H5:E300C, and H5:R303C all exhibited a transport activity below 10% of that of the wild-type. The double cysteine mutant H1–H3:D99C/K201C was inactive similarly to its single mutant H1:D99C; despite that, H3:K201C had 38% activity with respect to the wild-type Ndt1p. H3–H5:W198C/R303C was also inactive similarly to both its corresponding single mutants, whereas H5–H1:E300C/K102C retained 22% transport activity unlike both its single mutants which had an activity below 10% compared to that of the wild-type. Swapping the residues of the latter charged pair as in H5–H1:E300K/K102E completely inactivated the carrier. These results suggest that (i) K201 is not crucial for transport, (ii) the salt bridge between K102 and E300 is not essential for transport, (iii) leaving each of K102 and E300 as an unpaired charged residue is deleterious for transport activity, and (iv) the positions of the charged residues H5-H1:E300/K102 are important.

## 3. Discussion

**Figure 5:**
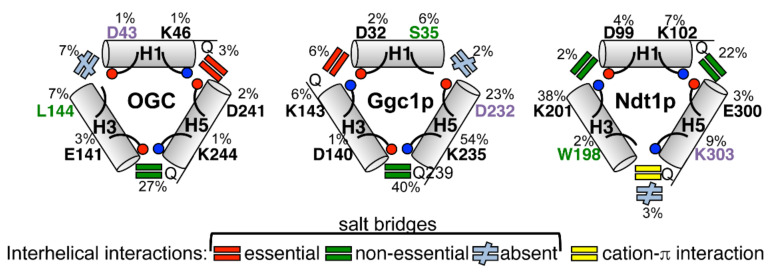
Schematic presentation of the M-gate salt bridge networks of OGC, Ggc1p, and Ndt1p. The positive and negative charges of the PX[DE]XX[KR] motifs are indicated by blue and red circles, respectively. Non-charged residues are in green and unpaired charges are in violet. Glutamines potentially forming glutamine braces of the salt bridge in proximity to the charged residues are indicated with Q.

In this study, the M-gates of MCs with one missing salt bridge (OGC, Ggc1p, and Ndt1p) were investigated by mutagenesis of the charged residue positions of the PX[DE]XX[KR] motifs and transport activity assessment, and its main results are summarized in Figure 5.

The results presented above and obtained using single cysteine mutations of the charged residue positions in the three PX[DE]XX[RK] motifs of OGC, Ggc1p, and Ndt1p show that in these three carriers most residues of the M-gate salt bridges are crucial for activity, but some are not. In fact, Ggc1p H5:D232C and H5:K235C, and Ndt1p H3:K201C have above 20% activity with respect to those of Cys-less Ggc1p and wild-type Ndt1p, respectively (Figure 2B, Figure 3B, and Figure 4B). This finding agrees with previous observations on the transport activity of the M-gate salt bridges-forming residues in the PX[DE]XX[RK] motifs of other MCs. In Mir1p and Ctp1p, all mutants neutralizing the charges of the PX[DE]XX[RK] motifs displayed a transport activity below 3% of that of the wild-type [46,47]. In contrast, in Aac2p H1:E45G and H3:R152A and in CAC H1:D32A and H5:K234A displayed over 20% transport activity compared to that of the wild-type [48,49]. It is noteworthy that Mir1p, Aac2p, and CAC have all the charged residues of the PX[DE]XX[RK] motifs in place, whereas Ctp1p and the three carriers investigated in this work miss one of these charged residues, meaning that non-essential residues are found in carriers with or without a complete salt bridge network. Taken together, these data show that in some MCs, such as OGC, all charged residues of the M-gate PX[DE]XX[RK] motifs are crucial for transport, whereas in other carriers, such as Ggc1p and Ndt1p, some of them facilitate transport without being indispensable. Given that the latter residues are different in the various transporters, it is likely that they are related to the specific MC and its substrate(s) rather than to general mechanistic/structural reasons common to all members of the MC family.

Because the M-gate PX[DE]XX[RK] motifs are part of the highly conserved signature motif sequence of MCs, they were not generally thought to have other functional roles than forming salt bridges. However, the absence of one or more of the charged residues at different positions in the M-gate PX[DE]XX[RK] motifs of OGC, Ggc1p, Ndt1p, and other MCs may be the result of particular requirements for substrate binding or the substrate-carrier binding energies triggering the conformational changes of the transporter. It was previously observed that the leucine (L144) in place of [RK] in the second PX[DE]XX[RK] motif of OGC is absolutely conserved in the OGC MC subfamily and cannot be replaced by C, K, R, I, or V without loss of activity, suggesting that leucine in this position has been selected by evolution [30]. In this work, we have shown that the cysteine replacement of both S35, in the [RK] position of the first PX[DE]XX[RK] motif of Ggc1p, and W198 in the [DE] position of the second PX[DE]XX[RK] motif of Ndt1p diminished transport activity almost completely (Figure 3B and Figure 4B). Serine in the position of S35 is conserved among homologues of Ggc1p and the tryptophan W198 in Ndt1p is conserved in the NAD^+^ carrier subfamily homologues as well as in MCs transporting pyrimidine nucleotides, FAD/folate, and coenzyme A in mitochondria, and NAD^+^/FAD in peroxisomes [13,14,50,51,52,53,54,55,56,57,58,59]. In these carriers, the tryptophan in the second repeat may form a cation-π interaction with [RK] of the third motif (Figure 4A) [32]. This tryptophan-dependent interaction may modulate the strength of the matrix network by reducing the interaction energy required for M-gate opening. There are other less conserved variations of the charged residues in the PX[DE]XX[RK] motifs in several MCs: [QNAT] instead of [DE] in the third PX[DE]XX[RK] motif of FAD/folate carriers and ATP-Mg^2+^/phosphate carriers [60,61,62]; [NM] instead of [RK] in the second PX[DE]XX[RK] motif of the dicarboxylate carriers [63,64,65]; [FY] instead of [DE] and [LM] instead of [RK] in the second and third PX[DE]XX[RK] motifs of the yeast oxaloacetate carriers, respectively [66,67]; and [VL] instead of [RK] in the third PX[DE]XX[RK] motif of the phosphate carriers [68,69]. The results presented here for OGC1, Ggc1p, and Ndt1p, therefore, provide evidence that evolution has selected the substitution of specific charged residues in the PX[DE]XX[RK] motifs of certain MCs for reasons connected to their specific substrates: either for direct binding to the substrate or for optimizing the binding energy required for the conformational changes involved in the transport cycle.

Surprisingly, not only the natural substitutions of specific charged residues in the PX[DE]XX[RK] motifs did not tolerate their mutation into cysteine (L144C of OGC, S35C of Ggc1p, and W198C of Ndt1p), as discussed in the previous paragraph, but also the replacement of their unpaired charged partners into cysteine, i.e., D43C of OGC, D232C of Ggc1p and R303C of Ndt1p, caused markedly decreased activity; almost completely in D43C of OGC and R303C of Ndt1p, and to 23% of the Cys-less version in D232C of Ggc1p (Figure 2A,B, Figure 3A,B and Figure 4A,B). This means that the unpaired charged residues in these positions of OGC, Ggc1p, and Ndt1p, which are obviously not involved in the interhelical M-gate salt bridge formation and not in glutamine brace interactions either (Figure 5), have other crucial roles in the transport mechanism of their respective MCs. As mentioned above, H3:R303 of Ndt1p may be necessary for a cation-π interaction with H2:W198. The unpaired charged residues of the OGC (D43) and Ggc1p (D232) M-gates, which are both negatively charged, may be involved in the destabilization of the respective salt bridge networks by coming in proximity to the negative charges of the substrate of OGC and Ggc1p (2-oxoglutarate and GTP/GDP, respectively) in a step of the transport mechanism following the binding of the substrate to the central binding site and before the carrier undergoes the transition from the C- to the M-state. Alternatively, the unpaired negatively charged residues of the OGC and Ggc1p M-gates may play a role in the charge relay switch [32] by attracting the positively charged residue of the PX[DE]XX[RK] motif on the same α-helix, when the interhelical salt bridges are destabilized upon substrate binding, and thereby facilitating the disruption of the salt bridge network and M-gate opening.

The data obtained using double cysteine substitutions of the charged residues of the three investigated MCs PX[DE]XX[RK] motifs show that the salt bridges H5–H1:D241/K46 of OGC and H1–H3:D32/K143 of Ggc1p are clearly essential for transport because all single and double cysteine mutants of these residues exhibited nearly no activity (Figure 2B, Figure 3B and Figure 4B). Again, surprisingly, the salt bridges OGC H3-H5:E141/K244, Ggc1p H3–H5:D140/K235, and Ndt1p H5–H1:E300/K102 are non-essential as their double cysteine mutants displayed substantial activity. In addition, the salt bridge Ndt1p H1-H3:D99/K201 is non-essential, as one of its single cysteine mutants (H3:K201C) showed high transport activity (Figure 2B, Figure 3B, and Figure 4B). The activity of this latter single mutant (Ndt1p H3:K201C) cannot be explained by the salt bridge being partly compensated by an interhelical H-bond between H1:D99 and H3:Q of the glutamine brace (Figure 5) simply because there is no glutamine in this position but a methionine (M205, Figure 1). The only single cysteine mutant with a high activity that might be explained by such a compensating interhelical interaction of a lacking salt bridge is Ggc1p H5:K235C. In fact, an H-bond between H3:D140-H5:Q239 can be envisaged. These results, showing that the matrix salt bridges of MCs may be essential or non-essential for transport function, demonstrate that they do not contribute equally to closing and opening the M-gate. Both these salt bridge types are found in different positions in the three MCs studied and therefore, once again, these conclusions reflect a specific relationship between the particular MC and its substrate(s). In addition, our proposal that, in at least some MCs, the charged residues of the PX[DE]XX[RK] motifs are involved in interactions with the substrates is substantiated by available data from molecular dynamics and docking studies of Ctp1p, SLC25A4, Aac2p, and SLC25A29 [47,70,71,72]. These data provide evidence that the substrates interact with the charged residues of the PX[DE]XX[RK] motifs and, in particular, with K37 and K239 (Ctp1p), K32 and D231 (SLC25A4), K33 and R236 (Aac2p), and D23, K26, E114, and D211 (SLC25A29).

Interestingly, the double mutants of the non-essential M-gate salt bridges H3-H5:E141C/K244C of OGC and H5–H1:E300C/K102C of Ndt1p displayed much higher activity compared to those of their corresponding single cysteine mutants, but not as high as Cys-less OGC and Ndt1p wild-type, respectively (Figure 2B and Figure 4B). This phenomenon of a transporter being active when an internal salt bridge might form (Cys-less OGC and Ndt1p wild-type) or not form (double cysteine mutants), or being inactive when each single charge is left unpaired, is indicative of a salt bridge that rearranges upon substrate binding [73]. Furthermore, swapping the two charged pair residues as in OGC H3-H5:E141K/K244E, Ggc1p H3-H5:D140K/K235D, and Ndt1p H5-H1:E300K/K102E led to a remarkably reduced activity (Figure 2B, Figure 3B, and Figure 4B). Therefore, the positions of these charged residues, i.e., the polarity of these salt bridge interactions, are important for “full” activity, most likely for their role in the charge relay. Considering these data together, we propose that the salt bridges are rearranged upon substrate binding, as expected from the C- and M-state structures, probably by a charge relay mechanism. This proposal is supported by the conclusions made in studies with lactose permease, which is also a membrane transporter but it belongs to a different protein family than MCs, based on the transport activities of double and single mutants of salt bridge-forming charged residues [73].

The sulfhydryl inhibition experiments on the OGC and Ggc1p double cysteine mutants (Figure 2C and Figure 3C,D) show that fairly large reagents (MTSES, m-PDM, and o-PDM) react with at least one cysteine, totally inhibiting the transport activity by obstructing the substrate translocation pathway. In contrast, PAO and diamide presumably react with both cysteines only in the C-state when these residues are in proximity to each other in the M-gate. These latter observations indicate that, when one of the salt bridges of the M-gate is substituted by cysteines connected through a covalent disulfide bond, transport is inhibited, as one might expect when the carrier is locked in the C-conformation. 

In conclusion, this study on the functional relevance of the charged residue positions in the M-gate PX[DE]XX[RK] motifs of OGC, Ggc1p, and Ndt1 shows that most residues in these positions, but not all of them, are crucial for function (see Figure 5). The salt bridges identified as essential, non-essential, or absent (due to a charged residue substituted with an uncharged one), as well as the positions of the evolutionary-selected uncharged residues of the PX[DE]XX[RK] motifs and their unpaired charged residues, are located differently in the structural models of the three MCs investigated. The findings of this investigation indicate that the evolutionary-selected uncharged residues and the unpaired charged residues of the M-gate PX[DE]XX[RK] motifs have important functional roles, which are different from forming salt bridges and are specific to the carrier and its substrate(s), such as interacting with the substrate and/or contributing to the binding energy driving the substrate-triggered stabilization/destabilization of the M-gate. Furthermore, the discovery of essential and non-essential salt bridges demonstrates that these interactions do not contribute equally to the transport of the substrate, which would not have been expected if the role of the charged residues were only the formation and breaking of salt bridges as part of the closing and opening of the M-gate. The results of the single unpaired charged residue mutations and the polarity of the non-essential salt bridges show that the positions of the charged residues are important and imply the involvement of a charge relay mechanism in the disruption of the interhelical network of salt bridges in the M-gate opening. In addition, the present overall picture of the relevance of the charged residue positions of the M-gate PX[DE]XX[RK] motifs, as deduced from mutagenesis studies of OGC, Ggc1p, and Ndt1p, may be extrapolated to other MCs with one or more charged residue missing. Our data also point to the possibility that some of the salt bridges of the many MCs having complete charged networks are non-essential and their residues fulfill other roles during the catalytic transport cycle, interacting with other residues within the protein structures to facilitate the stabilization or destabilization of their M-gates. Future studies are needed to address these latter two issues.

## 4. Materials and Methods

### 4.1. Materials

Designed oligonucleotides and DNA extraction kits (product codes: 27104 and 28104) were ordered from Qiagen. Expand High fidelity Taq polymerase (11732641001), T4 DNA ligase (10716359001), HindIII (10656321001), and BamHI (10 656 275 001) were from Roche Diagnostics (Mannheim, Germany). NdeI (R0111S) was from New England Biolabs and the dNTP mix (18427013) from Invitrogen. Tryptone (211921), yeast extract (212750), and sodium (2-sulfonatoethyl)-methanethiosulfonate (MTSES, AM3720 INTERCHIM) were from Thermo Fisher. Amberlite XAD-4 (06444), ampicillin (A9518), bathophenanthroline (146617), diamide (D3648), dithioerythritol (D8255), egg yolk phospholipids (lecithin from eggs, 61755), Na_2_SO_4_ (239313), N-dodecanoylsarcosine (sarkosyl, L512), N,N′-1,2-phenylenedimaleimide (o-PDM, 104590), N,N′1,3-phenylenedimaleimide (m-PDM, 160458), PIPES (P6757), phenylarsine oxide (PAO, P3075) pyridoxal-5’-phosphate (82870), sephadex G-75 (17-0050-01), and Tris base (10708976001) triton X-114 (93422) were from Sigma-Aldrich. The radioactive substrates 2-Oxo [1-^14^C]glutaric acid (NEC597) and ^14^C-NAD^+^ (NEC831) were purchased from Perkin Elmer, and [8-^3^H]GTP (TRK314) from Amersham Biosciences. All reagents were of analytical grade.

### 4.2. Construction of Plasmids and Site-Directed Mutagenesis

The DNA sequences of the Cys-less OGC and Ggc1p as well as of wild-type Ndt1p were used as templates to generate single and double cysteine-replacement mutants, as described previously [40]. All mutations were introduced by the overlap extension PCR method [74], using oligonucleotides with appropriate mutations in their sequences as well as 5’- and 3’-flanking primers with the following restriction sites: NdeI and HindIII for Cys less OGC; BamHI and HindIII for both Cys-less Ggc1p and wild-type Ndt1p. The restriction-digested PCR products were inserted into the expression vector pMW7 by T4 DNA ligase and transformed into *E. coli* DH5α cells. Transformants, which were selected on 2 x TY plates containing ampicillin (100 μg/mL), were screened by direct colony PCR and by restriction digestion of the purified plasmid DNA. All mutant constructs were verified by DNA sequencing.

### 4.3. Overexpression and Purification of the Recombinant MC Proteins

The proteins were overproduced as inclusion bodies in the cytosol of *E. coli*, as described previously [10,13,14,30,41,75]. The inclusion bodies were purified by sucrose layer density gradient centrifugation [10] and washed at 4 °C with TE buffer (10 mM Tris-HCl, pH 8.0, and 1 mM EDTA). Then they were washed twice with a buffer containing 10 mM PIPES, pH 7.0, 3% (*w/v*) Triton X-114, 1 mM EDTA, and 20 mM Na_2_SO_4_, and finally with TE buffer again. The recombinant proteins were solubilized in a buffer containing 2.5% (*w/v*) sarkosyl, 1 mM EDTA, and 10 mM Tris-HCl pH 7.0. The residual material was removed by centrifugation (258,000 g for 1 h at 4 °C).

### 4.4. Reconstitution of the Purified MC Proteins into Liposomes and Transport Measurements

The solubilized recombinant proteins in sarkosyl were reconstituted into liposomes in the presence of substrates by the cyclic removal of the detergent with a hydrophobic column of Amberlite beads, as described previously [10,13,14,30,41,75]. The external substrate was removed from proteoliposomes on Sephadex G-75 columns. Transport at 25 °C was started by adding radiolabeled substrate to eluted proteoliposomes and terminated by the addition of 20 mM pyridoxal 5’-phosphate and 16 mM bathophenanthroline, which, in combination, inhibit the activity of several mitochondrial carriers completely and rapidly [76,77,78,79]. In controls, the inhibitors were added at the beginning together with the labeled substrate according to the “inhibitor stop” method [75,80]. The external radioactivity was removed on Sephadex G-75 columns and the radioactivity in the proteoliposomes was measured. The experimental values were corrected by subtracting control values. The initial transport rates were calculated from the radioactivity taken up by proteoliposomes after 30 s, i.e., in the initial linear range of substrate uptake, and were corrected by taking into account the efficiency of reconstitution, i.e., the proportion of reconstituted protein [7,8,65,81]. To investigate the effect of sulfhydryl reagents on double cysteine-replacement mutants, proteoliposomes were preincubated in the presence or absence of 5 mM MTSES, 2 mM diamide, 2 mM o-PDM, 2 mM m-PDM, or 1 mM PAO for 10 min at 25 °C with or without 10 mM DTE (in the case of PAO and diamide). After removal of unbound reagents by Sephadex G-75 chromatography, transport was initiated by adding radiolabeled substrate and terminated after 30 s.

### 4.5. Additional Experimental Methods

SDS-PAGE for all expressed and purified mutant proteins was performed as described previously [7]. The amount of pure recombinant proteins was estimated from Coomassie Blue-stained SDS-PAGE gels with the Bio-Rad GS-700 Imaging Densitometer (Bio-Rad Laboratories, San Francisco, CA, USA) using carbonic anhydrase as a protein standard [82]. The extent of incorporation of recombinant proteins into liposomes was determined as described [83], except that protein concentration was determined by laser densitometry of stained SDS gels after extraction of lipid by organic solvents [82].

## Data Availability

Not applicable.

## Abbreviations

AAC: ADP/ATP carrier; C-gate: cytoplasmic gate; C-state: cytoplasmic state; DTE: dithioerytritol; EPRA: expression, purification, reconstitution and transport assay; Ggc1p: GTP/GDP carrier; MC: mitochondrial carrier; M-gate: matrix gate; m-PDM: N,N′1,3-phenylenedimaleimide; M-state: matrix state; MTSES: sodium (2-sulfonatoethyl)-methanethiosulfonate; Ndt1p: NAD^+^ transporter; OGC: 2-oxoglutarate carrier; o-PDM: N,N′-1,2-phenylenedimaleimide; PAO: phenylarsine oxide; PIPES: 1,4-piperazine diethanesulfonic acid.
